# Flavonoid allosteric modulation of mutated visual rhodopsin associated with retinitis pigmentosa

**DOI:** 10.1038/s41598-017-11391-x

**Published:** 2017-09-11

**Authors:** María Guadalupe Herrera-Hernández, Eva Ramon, Cecylia S. Lupala, Mercè Tena-Campos, Juan J. Pérez, Pere Garriga

**Affiliations:** 1 Grup de Biotecnologia Molecular i Industrial, Centre de Biotecnologia Molecular, Departament d’Enginyeria Química, Universitat Politècnica de Catalunya, Edifici Gaia, Rambla de Sant Nebridi 22, 08222 Terrassa, Catalonia Spain; 2 Laboratori d’Enginyeria Molecular, Departament d’Enginyeria Química, Universitat Politècnica de Catalunya, Barcelona Tech, Barcelona, Catalonia Spain; 3 Present Address: Campo Experimental Bajío, INIFAP, Km 6.5 Carretera Celaya-San Miguel de Allende, Celaya Gto México, CP 38110 Mexico

## Abstract

Dietary flavonoids exhibit many biologically-relevant functions and can potentially have beneficial effects in the treatment of pathological conditions. In spite of its well known antioxidant properties, scarce structural information is available on the interaction of flavonoids with membrane receptors. Advances in the structural biology of a specific class of membrane receptors, the G protein-coupled receptors, have significantly increased our understanding of drug action and paved the way for developing improved therapeutic approaches. We have analyzed the effect of the flavonoid quercetin on the conformation, stability and function of the G protein-coupled receptor rhodopsin, and the G90V mutant associated with the retinal degenerative disease retinitis pigmentosa. By using a combination of experimental and computational methods, we suggest that quercetin can act as an allosteric modulator of opsin regenerated with 9-*cis*-retinal and more importantly, that this binding has a positive effect on the stability and conformational properties of the G90V mutant associated with retinitis pigmentosa. These results open new possibilities to use quercetin and other flavonoids, in combination with specific retinoids like 9-*cis*-retinal, for the treatment of retinal degeneration associated with retinitis pigmentosa. Moreover, the use of flavonoids as allosteric modulators may also be applicable to other members of the G protein-coupled receptors superfamily.

## Introduction

G protein-coupled receptors (GPCRs) superfamily is the largest family of signal transduction molecules involved in most relevant physiological processes. They respond to a broad spectrum of chemical entities, ranging from protons and calcium ions to small organic molecules (including odorants and neurotransmitters), peptides and glycoproteins. They are activated as a result of the binding of extracellular receptor-specific ligands to their extracellular or transmembrane domains and transmit the resulting extracellular signals^1^.

These receptors are widely studied because of their potential use as pharmacological targets in drug development. The progress in X-ray crystallography has increased the number of GPCRs structures solved and unveiled precise snapshots of ligand-receptor interactions. Moreover, some receptors have been crystallized in different functional states in complex with antagonists, partial agonists, full agonists, biased agonists and allosteric modulators, providing further insights into the mechanisms of ligand induced GPCR activation^2^. Given the enormous diversity of the ligand binding pockets and the highly dynamic nature of GPCRs, a more detailed understanding of GPCR binding sites has proven valuable for meeting more refined requirements for drug design. Increased interest has been developed for ligands that bind to putative allosteric sites that can provide increased selectivity for GPCRs subtypes thus reducing undesired side effects^3^.

Rhodopsin (Rho), a prototypic member of the GPCRs superfamily and the first for which the crystal structure was solved^4^, is the major protein found in the disk membranes of the rod outer segment of retinal rod photoreceptor cells. It mediates dim light vision by converting photons into chemical signals that can trigger the biological processes enabling the brain to sense the light stimulus. Mutations in Rho are associated with retinitis pigmentosa (RP), a group of inherited visual diseases that cause retinal degeneration. These mutations can cause protein misfolding that leads to a progressive loss of rod and cone cells resulting in vision loss^5–7^. A number of experimental studies using RP mutants have been carried out in order to decipher the molecular mechanisms of the disease as a necessary step aimed at the development of novel treatments. Some of the proposed strategies to fight this condition are based on pharmacological rescue, in which small molecules known as chemical or pharmacological chaperones bind to and stabilize misfolded opsins^8^. Different compounds have been studied to counteract Rho mutations effects, like 11-*cis*-retinal (11CR)^9^, valproic acid^10^, 9-*cis*-retinal (9CR)^11^ and some antioxidants^12, 13^, among others. There is a renewed interest in the use of natural products in the sustained effort to discover new GPCR ligands. Given the interest in finding new ligands that can act as allosteric modulators and compensate the effects caused by RP mutations, in the present study we have evaluated the effect of quercetin (Q) on Rho and a mutant associated with RP. Q is one of the most often studied dietary flavonoids ubiquitously present in various vegetables, tea and red wine, and known for its potential beneficial effects on health^14^. For this purpose, WT Rho and the recombinant G90V mutant associated with RP were expressed in the presence of Q, regenerated with 11CR or 9CR, and the physico-chemical and functional properties of the purified receptors were evaluated.

We found that Q-treated G90V mutant opsin increased its chemical stability and its chromophore regeneration rate, and slowed down its MetaII decay process, when it was regenerated with 9CR. Also, a clear change in the kinetics mode of the G-protein transducin activation was observed in a functional assay. Moreover, *in silico* analysis confirmed the preference of Q for a binding site which is only present when opsin is regenerated with 9CR.

## Results

### UV-Vis spectroscopic characterization

**Figure 1:**
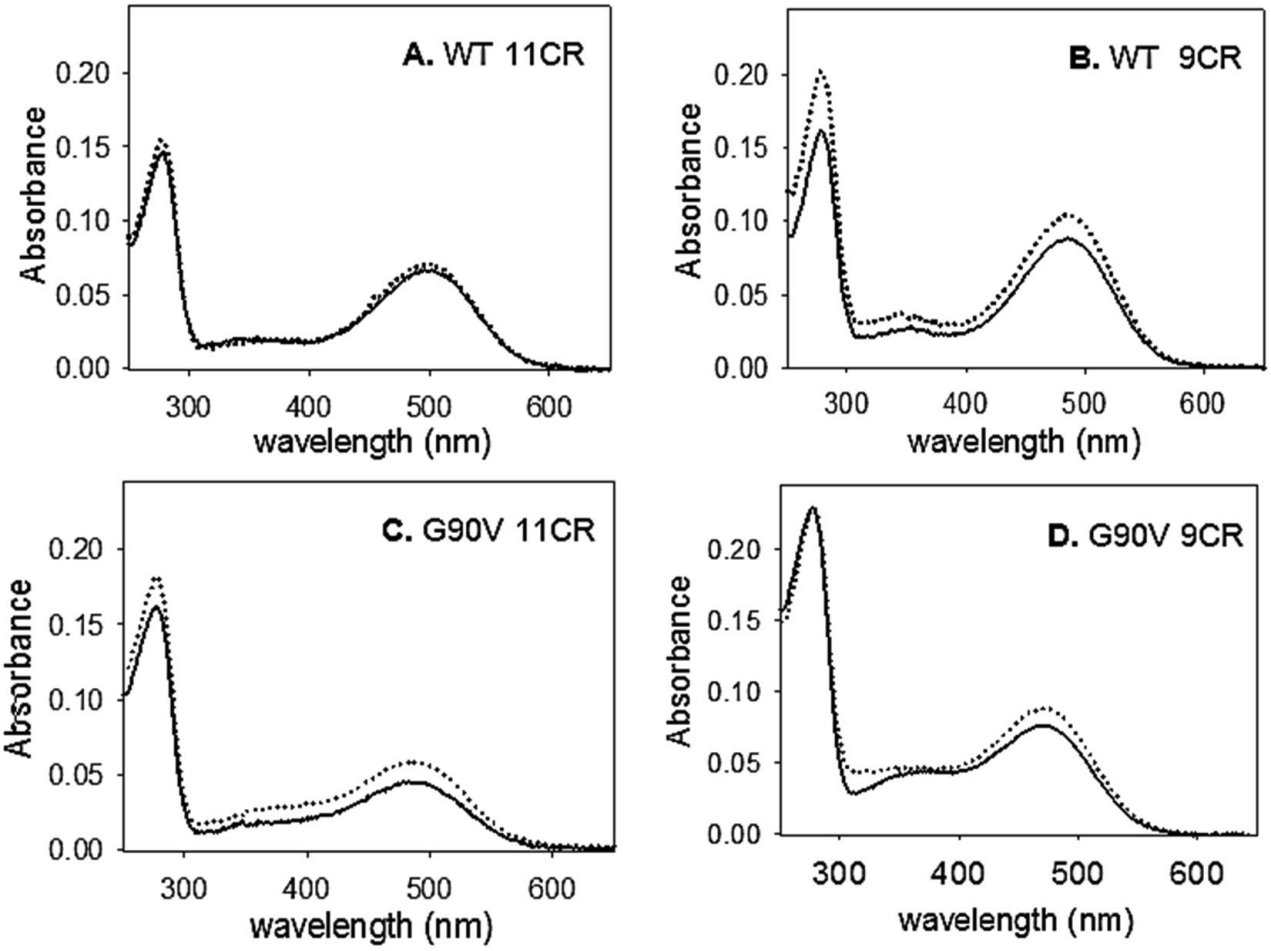
Absorption spectra of the first elution of immunopurified WT and G90V mutant regenerated with 11-cis-retinal (11CR) and 9-cis-retinal (9CR). After the immunopurification the receptors were characterized by UV-vis spectroscopy. Solid line represents the receptor without treatment, dotted line represents the receptor with 1 µM Q treatment. Samples were eluted with 100 µM of 9-mer peptide in PBS pH 7.4 and 0.05% DM. Spectra were recorded at 20 °C. (**A**) WT 11CR. (**B**) WT 9CR. (**C**) G90V 11CR. (**D**) G90V 9CR.

The UV-vis spectra of WT and G90V mutant receptors first elutions were recorded immediately after immunopurification (Fig. 1). WT Rho without (WT 11CR) and treated with 1 µM Q (WT 11CR-Q) showed similar spectroscopic patterns with a λ_max_ at 500 ± 1 nm as well as a similar A_280_/A_max_ ratio (Table 1). WT isorhodopsin (opsin regenerated with 9CR) showed a blue shift of 15 nm compared to WT 11CR which may be attributed to the decrease in bond length alternation of the retinal and its interaction with the amino acids in the binding pocket. In the case of G90V mutant (G90V 11CR) without Q treatment, a blue shift of about 10 nm was observed with respect to WT 11CR (Table 1), a behavior that has already been previously reported^15^. The G90V mutant with 1 µM Q treatment (G90V 11CR-Q) showed a similar Amax as G90V 9CR. A slightly increased A_280_/A_max_ ratio was previously reported for this mutant^15, 16^ that essentially agrees with the results obtained here for G90V 11CR. This increase could be due, at least partially, to the presence of a small fraction of misfolded (non-retinal binding) protein or to the lack of structural stability of the regenerated mutant protein. For this mutant, Q treatment reduced the ratio by 15% as well as increased its purification yield (Fig. 1C and Table 1). G90V 9CR showed a larger blue shift due to the combined effect of the mutation and the 9CR presenting a λ_max_ of 472 ± 3 nm that was similar to that of G90V 9CR-Q. In both cases, a higher purified protein yield was observed than with 11CR, and the absorbance ratio was more similar to the WT, especially in the case of the G90V 9CR-Q (Table 1). Western blot analysis indicated no major changes in the electrophoretic pattern of WT and G90V mutant expressed with and without 1 µM Q treatment (Fig. S1, Supplementary Information).

**Table 1:** Spectroscopic properties of WT regenerated with 9CR and 11CR with and without 1 µM Q treatment.

	λ_max_	Ratio A_280_/A_max_
WT 11CR	500 ± 1	2.1 ± 0.2
WT 11CR-Q	499 ± 2	2.2 ± 0.3
WT 9CR	485 ± 3	1.9 ± 0.2
WT 9CR-Q	486 ± 2	1.9 ± 0.3
G90V 11CR	489 ± 2	3.7 ± 0.2
G90V 11CR-Q	488 ± 2	3.1 ± 0.2*
G90V 9CR	472 ± 3	2.9 ± 0.2
G90V 9CR-Q	471 ± 2	2.5 ± 0.2*

λmax, mean value ± SE of the visible peak of samples obtained in independent purifications (n = 3). The ratio absorbance at 280 nm to A_max_ provides a measure of both sample purity and the ability of opsin to form pigment with the chromophore. Data are mean ± SE of the visible peak of samples obtained in independent purifications (n = 3). Statistical significance due to the Q treatment was marked with asterisks (*).

**Figure 2:**
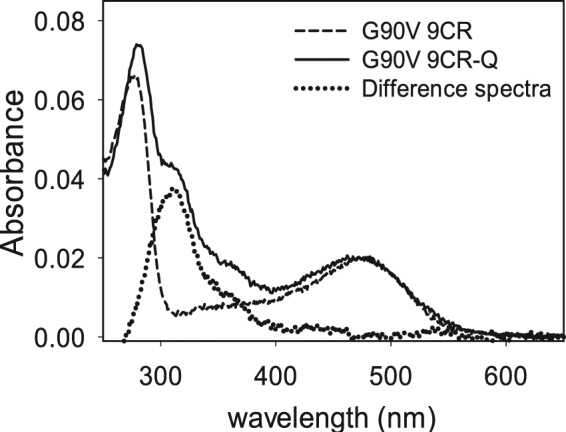
Q is identified in the third elution of immunopurified G90V 9CR mutant sample. During the experiments, a second protein elution was performed to recover as much protein as possible. This second elution was done in PBS pH 6 and used in the regeneration experiments. When it was possible, up to a third elution was carried out in the samples that showed a higher yield, as was the case in the treatments with Q in the receptors regenerated with 9CR. Solid line represents the third elution of G90V 9CR without Q treatment. Dashed line represents the third elution of G90V 9CR-Q with treatment of 1 µM Q. Dotted line represents the difference spectra of G90V 9CR-Q minus G90V 9CR. Samples were eluted with 100 µM of 9-mer peptide in PBS pH 7.4 and 0.05% DM.

During the immunopurification an altered UV-vis spectrum of the third elution of G90V 9CR-Q could be observed (Fig. 2). Thus, when the absorption spectrum of the third elution of G90V 9CR-Q (performed at pH 6.0) was recorded, a different spectroscopic pattern was observed and two additional absorbance bands could be detected, one larger band at 310 nm and a small shoulder at 360 nm. In addition, the band a 280 nm representing the protein fraction showed a red shift of 4 nm (Fig. 2). Furthermore, Western blot of these samples was performed and the G90V 9CR-Q mutant showed reduced intensity in the band at about 27 KDa attributed to truncated protein (Fig. S2, Supplementary Information). The presence of Q in the purified sample was confirmed by means of HPLC-ESI-MS/MS analysis (Fig. S3, Supplementary Information).

### Photobleaching and acidification

**Figure 3:**
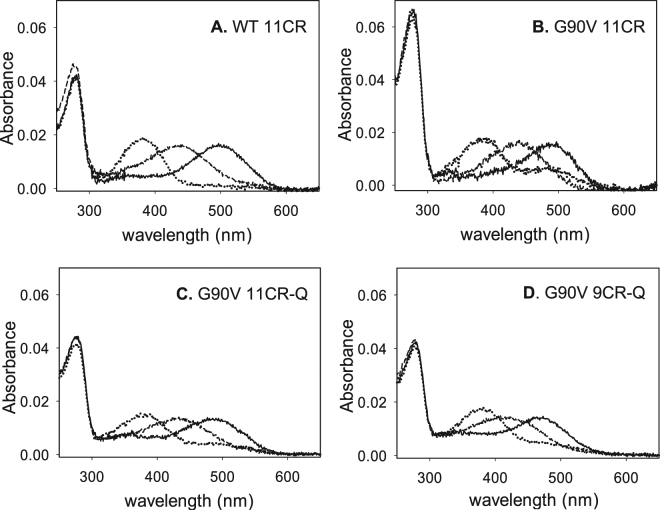
UV-vis characterization of WT 11CR, G90V 11CR with and without Q treatment and G90V 9CR-Q. Dark state (solid line), photobleaching (dotted line) and acidification (dashed line). Samples in PBS pH 7.4 and 0.05% DM. Spectra were recorded at 20 °C. (**A**) WT 11CR. (**B**) G90VT 11CR. (**C**) G90V 11CR-Q. (**D**) G90V 9CR-Q.

Photobleaching of Rho can be followed by the blue-shift of the 500 nm chromophoric band in the visible region to 380 nm. This shift is due to the Schiff base (SB) nitrogen deprotonation in the Metarhodopsin II (Meta II) state^17^. The UV-vis spectra of WT (Fig. 3) and G90V mutant (Fig. 3) were recorded in the dark, upon photobleaching for 30 s and after subsequent acidification.

The main difference observed was in the case of G90V 11CR sample (Fig. 3B) which upon illumination did not show a complete conversion of the visible band to the 380 nm species when compared to the WT spectrum (Fig. 3A). This remaining band (about 44% of the dark visible band) had a similar visible wavelength maximum as the dark pigment, suggesting conversion to a photointermediate with a retinal binding pocket similar to the dark pigment, including the presence of a protonated SB (PSB) linkage^18^. This band was diminished by the presence of Q since, in G90V 11CR-Q (Fig. 3C), the remaining band upon photobleaching was only about 30% of the initial visible absorbance band.

The change of the natural chromophore to the 9CR analog reduced the effects observed in the remaining Amax band that was only 30% as opposed to 44% for G90V-11CR. In this case, however, the presence of Q did not further affect the intensity of this band. (Figure 3D). No changes were observed in photobleaching pattern of the other receptors.

For all samples, a slightly higher A_380nm_ with regard to the A_max_ in the visible region was observed after illumination. The acidification of these photoactivated receptors resulted in a distinct behavior, a band with a maximal absorbance at 440 nm corresponding to the PSB could be observed in all cases except in the case of G90V 9CR-Q which showed an asymmetric band with an absorbance maximum at 415 nm after acidification (Fig. 3D). This behavior was previously reported for some Rho mutants^15, 19^ but in this case it may be presumably due to the presence of Q and not to the mutation itself. The band obtained by acid denaturation of illuminated G90V 9CR-Q indicated that the SB linkage had undergone partial hydrolysis and this band would show contributions from both free retinal at 380 nm and PSB-linked species absorbing at about 440 nm.

### Thermal stability

The thermal stability of the proteins was determined by measuring the UV-vis spectra of the samples from 250 nm to 650 nm with time at the temperature of 48 °C. It has been shown that Rho can be activated in the dark by increasing the temperature that would force chromophore isomerization^20^. In the thermal stability assay, the G90V mutant was very unstable in the dark state compared to the WT, showing faster thermal bleaching with t_1/2_ of ~2 min (Table 2). In the WT, the change of the natural chromophore to the 9CR analog reduced significantly its thermal stability by 30%, an effect that was compensated by Q. In the case of G90V-9CR, Q apparently increased the thermal stability of the mutant although this difference was found not to be statistically significant. In any case, the potential stabilizing effect of Q on the G90V-9CR mutant would be difficult to detect due to the fact that the temperature-induced retinal isomerization and hydrolysis of the deprotonated SB is very fast due to the intrinsic thermal instability of the G90V mutant.

**Table 2:** Thermal stability of the immunopurified WT and G90V mutant regenerated with 11CR and 9CR.

	W/O Q t_1/2_ (min)	Q 1 µM t_1/2_ (min)
WT 11CR	82.0 ± 2.2	79.2 ± 5.9
WT 9CR	50.0 ± 2.8	70.0 ± 5.0*
G90 V 11CR	2.0 ± 0.14	1.79 ± 0.13
G90V 9CR	1.86 ± 0.11	2.25 ± 0.21

WT and G90V mutant were regenerated with 11CR or 9CR and immunopurified in PBS pH 7.4 and 0.05% DM, and were incubated at 48 °C. The normalized Abs values at λ_max,_ in the visible region, were plotted as a function of incubation time and the t_1/2_ was calculated. A. WT. B. G90V. W/O = without. Mean value ± SE obtained in independent purifications (n = 3). In our study, only the differences observed in the sample of WT 9CR(*) with Q treatment was found to be statistically significant.

### Chemical stability

**Figure 4:**
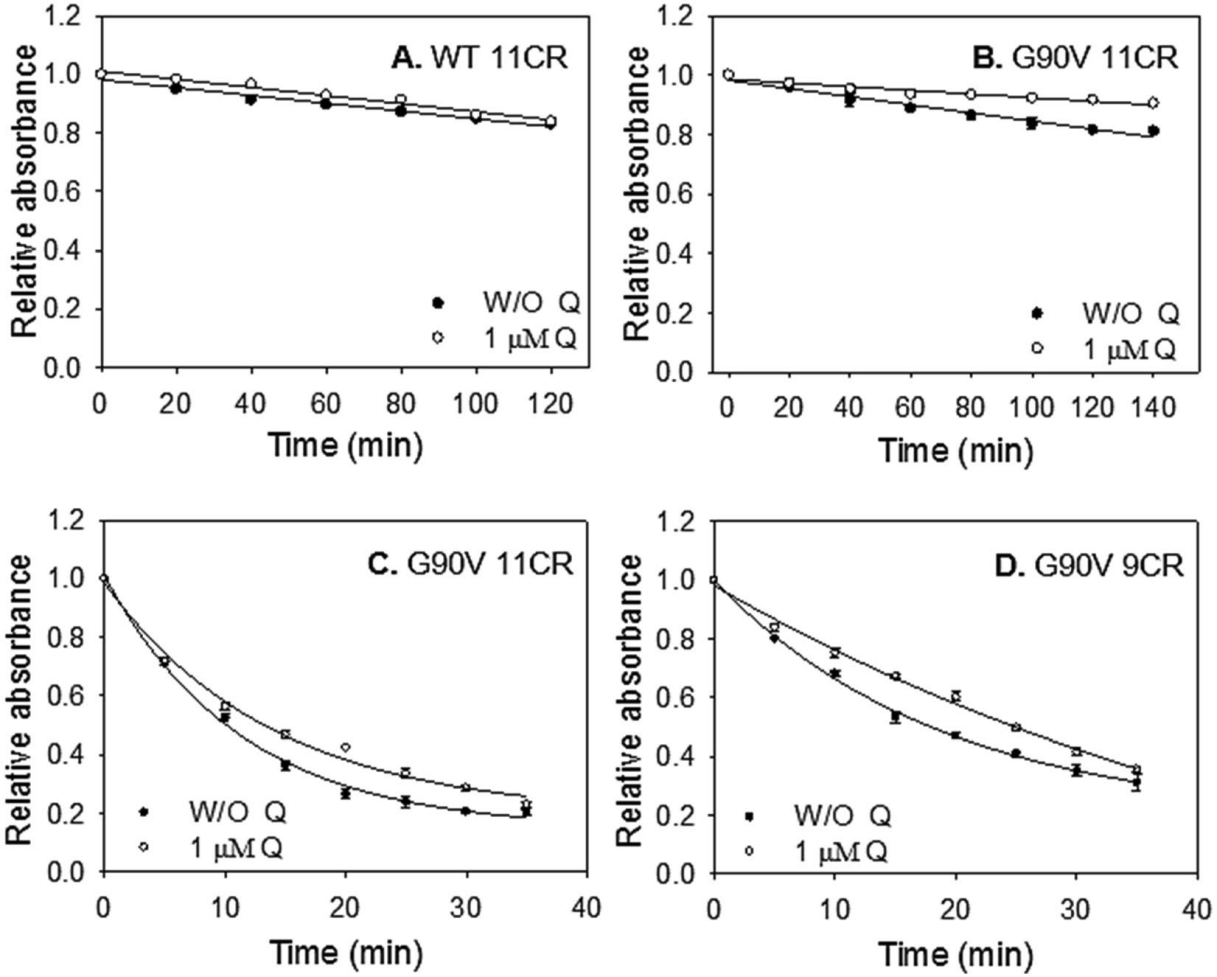
Chemical stability of the immunopurified WT and G90V mutant. Immunopurified protein samples in PBS pH 7.4 and 0.05% DM, after Q treatment, were incubated with 50 mM hydroxylamine, pH 7 and the decrease of A_max_ was recorded over time at 20 °C. (**A**) WT 11CR with (⚪) and W/O 1 µM Q (●). (**B**) WT 9CR with (⚪) and W/O 1 µM Q (●). (**C**) G90V 11CR with (⚪) and W/O 1 µM Q (●). (**D**) G90V 9CR with (⚪) and W/O 1 µM Q (●). Mean value and standard error (SE) obtained in independent purifications (n = 3).

**Figure 5:**
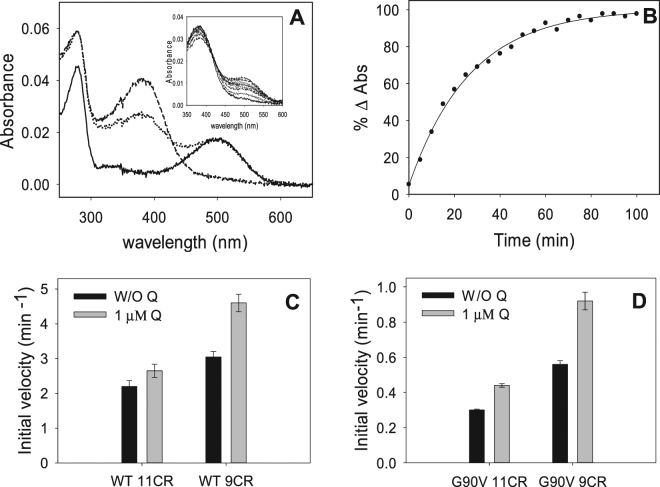
Initial rates of chromophore regeneration of the immunopurified WT and G90V mutant with 1 µM Q treatment. (**A**) 2.5 molar excess fold of 11CR or 9CR (0.925 µM) (dotted line) with regard to Rho concentration (0.37 µM) (solid line) was added to the immunopurified WT and the G90V mutant, in the different buffers containing 0.05% DM + 1 µM Q, samples were illuminated with light of >495 nm to avoid photobleaching of the free retinal (dashed line), and successive spectra were recorded at 20 °C in the dark until no further increase in A_max_ was detected (inset). (**B**) The normalized absorbance values at A_max_ were plotted as a function of time, the data were fit to a single exponential function and the initial rates derived. (**C**) Initial rates of chromophore regeneration of WT 11CR and WT 9CR. (**D**) Initial rates of chromophore regeneration of G90V 11CR and G90V 9CR. Mean value and standard error (SE) obtained in independent purifications (n = 3).

Hydroxylamine is a compound that is used in Rho studies to determine whether a mutation can affect the structural compaction around the SB environment. This reagent can enter the retinal binding site of photoactivated Rho (but not in its dark-state conformation) and hydrolyze the SB linkage. The WT 11CR was remarkably stable in the presence of hydroxylamine, as well as WT 9CR, and they showed a linear kinetics for this assay (Fig. 4A and B, open circles). For the WT 9CR, the presence of Q results in a slight decrease of hydroxylamine accessibility to the retinal binding site and hydrolysis of the SB linkage. In contrast, G90V 11CR showed a non-linear kinetics and a dramatic decrease due to the less compact structure in the SB linkage environment, as previously observed^15, 16^ (t_1/2_ = 10.2 ± 0.3). Here, Q slightly increased the chemical stability of G90V 11CR-Q (t_1/2_ = 13.3 ± 0.6). As for G90V, the 9CR analog increased the stability of this mutant (compare open circles in Fig. 5C and D) and also showed a non-linear kinetics with a t_1/2_ = 17.6 ± 0.8. A similar behavior was previously reported for this mutant^15^. Furthermore, treatment with Q on this mutant receptor further increased the chemical stability of the receptor (Fig. 5D), which is indicative of a more compact structure of the protein, at least in the vicinity of the chromophore SB linkage (t_1/2_ = 25.0 ± 0.6). Furthermore, this kinetic behavior was more linear as in the case of WT (compare Fig. 4D, open circles, and Fig. 4A and B).

### Chromophore regeneration

After Rho has been activated by light, it undergoes a series of inactivating reactions, passing through several intermediate forms before being regenerated to the original opsin state, and being able to bind a fresh 11CR molecule^21^. In specific cases where opsin fails to reunite with the chromophore to regenerate Rho, the persistent activation of G protein by opsin destabilizes and eventually damages the rod cells leading to retinal degeneration^22, 23^ Hence, the importance of the Rho regeneration process.

WT 11CR presented the lowest regeneration rate which was not affected by Q treatment (Fig. 5C). Replacing the natural chromophore with 9CR analog showed a significant increase of chromophore regeneration initial rate compared to WT 11CR. This rate was increased by 50% as a result of Q treatment (Fig. 5C). Regarding to G90V mutant, the presence of Q significantly increased the initial rate of regeneration in both G90V 11CR and G90V 9CR, having a greater effect on the mutant regenerated with 9CR (65% increase in the chromophore regeneration initial rate compared to the 45% increase obtained in the case of G90V 11CR-Q) treatment (Fig. 5D).

This result agrees with the amount of protein obtained after purification of these receptors where the highest yield was obtained in the G90V 9CR-Q case (see Fig. 1). This could be attributed to the stronger interaction energy acquired by the C-13 methyl group of 9CR from Y268 and W265, favoring the entry into the retinal binding site^24^.

### Meta II decay

The stability of the active state of purified WT and mutant was determined by means of fluorescence spectroscopy. In the dark state, Trp265 fluorescence is quenched by the β-ionone ring of the retinal and, upon illumination, retinal is released from the protein binding pocket thereby resulting in an increase in Trp265 fluorescence emission. This retinal release process (which closely parallels Meta II decay under our experimental conditions) can be followed at 330 nm for an excitation wavelength of 295 nm. The fluorescence changes were monitored continuously over time^25^. To determine the t_1/2_ values for retinal release, experimental data was analyzed using a mono-exponential rise to maxima fit.

**Figure 6:**
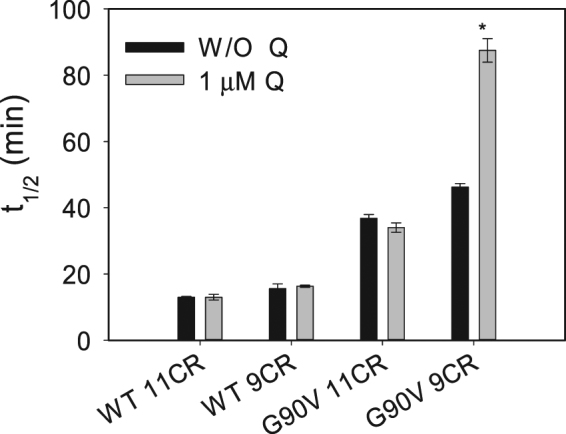
Meta II decay of the immunopurified WT and G90V mutant regenerated with 11CR or 9CR with or W/O 1 µM Q treatment. Samples were incubated at 20 °C, and after a steady base line was obtained, they were photobleached and the Trp fluorescence was monitored over time. The fluorescence increase was fit to a single exponential function and the t_1/2_ calculated. Mean value and standard error (SE) obtained from independent purifications (n = 3).

The Meta II decay process was slightly slower for WT 9CR than for WT 11CR (Fig. 6). In both cases, the t_1/2_ was not affected by Q. G90V 11CR mutant showed a higher difference compared to the WT with a t_1/2_ of 36 ± 1.1 which is twice slower. For this mutant, regenerated with its natural chromophore, the presence of Q did not affect the Meta II decay (Fig. 6).

A different behavior was observed when the G90V mutant was regenerated with 9CR analog in which the hydrolysis of the photointermediates Meta II was slower (46 min) compared to the G90V 11CR (36 min). Strikingly, in this case the presence of Q almost doubled the time needed for the Meta II decay with a t_1/2_ of 88 min.

### Transducin activation

**Figure 7:**
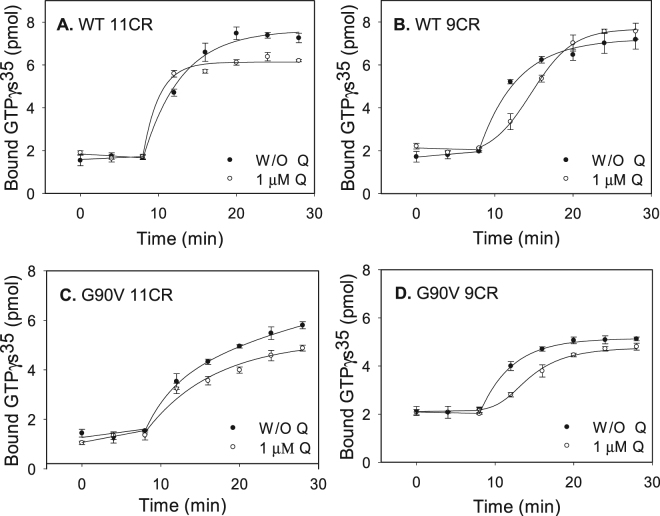
Gt activation by WT and G90V mutant regenerated with 11CR or 9CR with or W/O 1 µM Q treatment. Gt activity was measured by means of a radionucleotide filter-binding assay in Gt buffer. The reaction was initiated by the addition of the WT or mutant, and samples were filtrated at different times in the dark and after illumination. (**A**) WT 11CR with (⚪) and W/O 1 µM Q (●). (**B**) WT 9CR with (⚪) and W/O 1 µM Q (●). (**C**) G90V 11CR with (⚪) and W/O 1 µM Q (●). (**D**) G90V 9CR with (⚪) and W/O 1 µM Q (●). Mean value and standard error (SE) obtained in independent purifications (n = 3).

In this assay, transducin activation by WT and mutant was measured in the dark and after photobleaching. A similar kinetics behavior for WT 11CR and WT 9CR was observed (Fig. 7A). In the case of WT 11CR-Q the activation rate was slightly faster than in the case of WT 11CR. Surprisingly, the WT 9CR-Q sample showed a completely different kinetics to the hyperbolic kinetics presented by WT 9CR in the absence of Q. In this case the kinetics is sigmoidal which would reflect cooperative binding (Fig. 7B).

G90V 11CR showed a similar kinetics to that of WT 11CR but in this case the rate of transducin activation was slower compared to WT 11CR (compare Fig. 7A and C). This demeanor observed in the transducin activation for this mutant, correlates very well with the results obtained in the Meta II decay experiments where the all-*trans*-retinal release as a consequence of Meta II hydrolysis is slower with respect to WT 11CR. For G90V 9CR-Q the transducin activation was slower compared to the mutant without treatment. In the case of the mutant regenerated with the 9CR analog, the same effect as in the WT 9CR case could be observed. Of all treatments, the G90V 9CR-Q showed the lowest Gt activation and could be correlated with its high t_1/2_ in the Meta II decay assay.

Our results are consistent with Q being bound to Rho in the purified samples because the analyzed parameters only vary in the purified protein samples coming from Q-treated cells (see Figs 5, 6 and 7). It is plausible that Q binds during the folding and membrane insertion process of the protein, in the cell culture, because we could not detect any effect of Q when the compound was added to the protein sample after purification at the last step before analysis (data not shown). Furthermore, we washed thoroughly the sepharose resin before protein elution and could not detect Q in the final wash (data now shown). We also performed a concentration of the purified G90V sample and we found that the concentration factor was similar for Rho (A_280concentrated_/_A280before concentration_ = 2.0) and Q (A_375concentrated_/A_375before concentration_ = 1.8). This result suggests that Q is specifically bound to the protein but further studies will be needed to confirm this binding.

### Molecular modeling studies

In order to get further insight into the experimental results reported in this work, we carried out computational studies to identify prospective binding sites of Q to these receptors.

**Figure 8:**
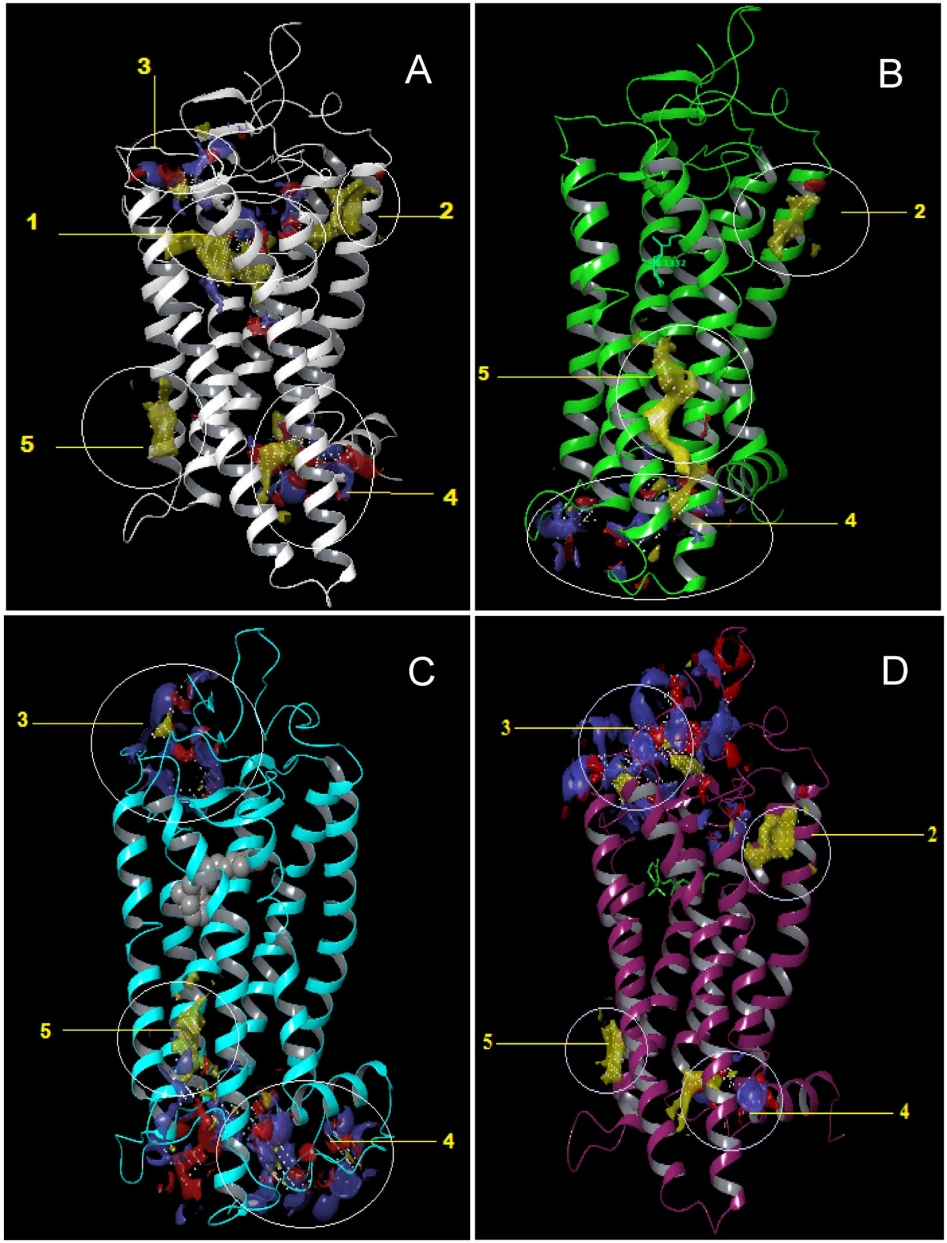
Pictorial view of the putative binding sites identified using SiteMap in: (**A**) opsin (**B**), rhodopsin (11CR), (**C**) isorhodopsin (9CR), and (**D**) Meta II.

Figure 8 shows pictorially identified prospective binding sites on opsin, 11CR bound to Rho and 9CR to Rho (9CR) using SiteMap. For opsin, five prospective binding sites have been identified (Fig. 8A). The first, annotated as 1 in the figure represents the orthosteric site, a pocket located inside the helix bundle just beneath the extracellular region of the receptor. Binding site number 2 is found just along the orthosteric site 1 but on the outside of helices TM1 and TM2. Site 3 is found on the extracellular region of the receptor and involves some residues in the ECL2 and some of N-terminal residues. Site 4 is found just above the intracellular region, on the side between TM3, TM4 and TM5. Lastly, site 5 is majorly located in the intracellular region of the receptor and it is the biggest site with both hydrophobic character and hydrogen bond acceptor and donor characteristics. Binding sites close to the intracellular side have been recently found in CCR2 and CCR9 chemokine receptors^26, 27^.

In the case of Rho with 11CR (Fig. 8B) or 9CR (Fig. 8C) site 1 is not available since it is already occupied by the corresponding retinal. Docking studies reveal three prospective sites for each complex previously identified in opsin. Specifically, sites 2, site 4 and site 5 were identified for 11CR, whereas site 3, site 4 and site 5 were identified for 9CR. Accordingly, these sites were the focus of subsequent docking studies. Results of these studies permitted to discard sites 2 and 5 that can be considered as shallow hydrophobic pockets, due to their nature. In contrast, Q was found to bind onto site 4 for 11CR and onto sites 3 and 4 in 9CR (Fig. S4, Supplementary Information).

We also conducted the corresponding docking of Q on the published crystal structure of photoactivated Rho, the Meta II structure. Interestingly, we find that in this structure the binding site 3 becomes available after the structural rearrangement ensuing receptor photoactivation (Fig. 8D).

## Discussion

9CR is the most studied analog of retinal that produces isorhodopsin containing a PSB between 9CR and opsin. It undergoes an identical bleaching sequence to that of Rho (regenerated with 11CR) and it is characterized by a blue-shifted A_max_ visible band. 9CR is often used as an artificial analog to probe the structure and function of native Rho^28^.

The pharmaceutical application of 9-cis retinoids to remedy retinal dysfunction caused by delayed or deficient regeneration with 11CR has been investigated over the past decade^11, 29–31^. Several properties have been attributed to this retinal analog such as the increase in stability of the RP mutant G90V^15^. Hence, these factors increase our interest in carrying out the current study using the 9CR analog. Furthermore, novel methodological approaches have been developed to try to control physiological processes by the use of light^32–34^. In this regard, optogenetic and opthopharmacological techniques are regarded as promising tools for the treatment of retinal degenerative diseases like RP. However, these techniques require the delivery of recombinant microbial opsins with potential immunogenic problems as in the case of optogenetics^35^ or chemical photochromic agents with potential toxicity as in the case of optopharmacology^36^. To overcome these drawbacks the use of natural products, like Q, alone or in combination with other molecules, like retinal analogs, can be a powerful strategy to counteract the effects of mutations associated with retinal degeneration in RP.

The results found in our study show the specific effect of Q on the binding properties of 9CR containing opsins, especially in the case of the G90V mutant in which its percentage and rate of regeneration were higher with this analog than with 11CR. This could be attributed to the stronger interaction energy acquired by the C-13-methyl group from Y268 and W265, favoring the entry into the retinal binding site^24^. In addition, its chemical stability also increases after regeneration with the retinal analog and that reflects an improvement of the structural compaction in the SB environment. Our results indicate a synergistic effect of the combination of 9CR and Q in improving the physico-chemical properties of RP mutant proteins. In this regard, besides the known pharmaceutical application of retinoids to address visual dysfunctions, other small molecules have also been investigated for their properties as pharmacological chaperones^8, 37, 38^. Many of these molecules would bind and stabilize mutant opsins thus improving folding problems^39^. Our results suggest that Q improved the folding and structural stability of the G90V RP mutant. In this case the A_280_/A_max_ ratio was more similar to the WT reducing in about 15% the presumed misfolding that is common for RP mutants^40^. Previous research suggested that flavonoids (the group to which Q belongs) may be involved in vision physiology and eye health^41^ but no clear proof was provided to date.

Our results suggest that Q may act as an allosteric modulator of Rho and G90V mutant when their orthosteric ligand is 9CR. *In silico* studies demonstrate that the potential ligand binding sites are different when the orthosteric ligand is 11CR or 9CR. The molecular docking results reveal that the binding site 3, which is not found in Rho, could potentially be the site where Q could bind. This site involves the ECL2 in which a slight difference was observed by superimposing the structures of Rho and isorhodopsin, difference that was also observed in the N-terminus. The second extracellular loop in particular has been the target of a number of functional studies indicating its role in GPCRs activation as a result of binding of either small molecules or large peptide ligands^42, 43^. In Rho, ECL 2 is part of the retinal plug^44^, and forms a cap over the binding site of its photoreactive chromophore. A well-defined H-bonded network stabilizes the ECL2 structure which is formed by a number of polar residues, with Glu181, at the center of this network, which is H-bonded to Tyr192 and Tyr268, and is connected to Glu113, the counterion to the retinal PSB. Computational studies identified ECL2 as part of the stable folding core of inactive Rho^45^. In its active conformation (Meta II), the displacement of ECL2 from the retinal binding site and a rearrangement in the hydrogen-bonding networks connecting ECL2 with the extracellular ends of TM4, TM5 and TM6, has been reported. Furthermore, NMR measurements revealed that structural changes in ECL2 are coupled to the motion of helix TM5 and breaking of the ionic lock that regulates activation^46^.

Given the characteristics of ECL2, it is possible that Q bound at this site can give more stability and compaction to the retinal binding pocket environment which is reflected in the improved chemical stability presented by WT 9CR-Q and G90V-9CR-Q. In addition, this more compact structure also affects the retinal release after the hydrolysis of Meta II which was increased to almost double in the G90V 9CR-Q mutant. This large difference in the Meta II decay for this mutant (with Q apparently bound at ECL2) would be probably due to the replacement of glycine by valine in the G90V mutant.

In the case of the photoactivated Meta II conformation, our molecular docking analysis indicates that site 3 becomes available for Q binding and this appears to be consistent with our experimental results. This suggests that binding site 3 is somehow only accessible during folding of the protein (as would happen when we treat COS-1 cells with Q) or after structural rearrangements associated with the formation of the active Meta II state. This finding is very interesting and opens up new avenues for exploring the interaction of Q with different Rho conformations with greater detail. All these results might be relevant for the different conformations adopted by other GPCRs.

In the transducin activation assays, very marked changes in the activation kinetics were observed again in the samples WT 9CR-Q and G90V 9CR-Q, and in the case of G90V 9CR-Q the transducin activation was somehow reduced compared to the mutant without Q treatment. These results suggest that the activation process is affected by Q, presumably bound to ECL2, altering the rearrangement in the hydrogen-bonding networks connecting this loop with the extracellular ends of TM5 impairing the breaking of the ionic lock that regulates activation^46^.

In conclusion, in this study, using various molecular biology techniques and analytical methods coupled with *in silico* computational studies, Q has been shown to act as an allosteric modulator of 9-cis-Rho and more importantly, that property has an effect on the stability of G90V 9CR mutant associated with RP. The results presented here demonstrate that the same allosteric modulator (Q) can act as an orthosteric ligand enhancer (because it increases the regeneration rate) and at the same time reduce transducin activation. This modulated response that presents Q like an allosteric modulator of Rho mutants can be exploited in drug design and the development of novel pharmacological approaches for RP treatment. These results open new possibilities to use natural polyphenolic compounds, in combination with specific retinoids like 9CR, for the treatment of retinal degeneration associated with RP. This approach will help in preventing potential immunogenic problems associated with the use of microbial opsins in optogenetic methods. It may also elude the potential toxic effects of photochromic ligands recently proposed as therapeutic agents in optopharmacological innovations. Furthermore this effect of Q as allosteric modulator may also be applicable to other members of the GPCRs superfamily.

## Experimental Procedures

### Materials

All chemicals were purchased from either Fisher or Sigma except were stated. 11CR was provided by National Eye Institute, National Institutes of Health (USA), mAb rho-1D4 antibody was purchased from Cell Essentials (Boston, USA) and was coupled to cyanogen bromide (CNBr)-activated Sheparose 4B beads. n-dodecyl-β-D-maltoside (DM) from Anatrace Inc. (Maumee, OH, USA), H-TETSQVAPA-OH peptide was synthetized by Unitat de Tècniques Separtatives i Síntesi de Pèptids (Barcelona, Spain), polyethyleneimine (PEI) was provided by Polysciences Inc. (USA), cellulose membrane and manifold for radioactivity assay was from Millipore, (France) and [S^35^]GTPγS was purchased from Merck. Dulbecco’s modified Eagle medium (DMEM) supplemented with fetal bovine serum, L-glutamine and penicillin-streptomycin was used to culture COS-1 cells. Q ≥ 95% (HPLC) was purchased from Sigma, Spain.

#### Buffers

The following solutions were used: buffer A (137 mM NaCl, 2.7 mM KCl, 1.5 mM KH_2_ PO_4_, and 8 mM Na_2_HPO_4_, pH 7.4) buffer B (0.05% DM in buffer A, pH 7.4) buffer C (0.05% DM in buffer A, pH 6), buffer Gt (25 mM Tris, pH 7.5, 100 mM NaCl and 5 mM magnesium acetate).

#### Cell line

COS-1 cells were obtained from the American Type Culture Collection (Manassas, Va).

### Methods

#### Construction, expression and purification of bovine recombinant wild type (WT) Rho and G90V mutant

WT and G90V opsins genes were constructed in the pMT4 plasmid vector by site-directed mutagenesis kit (QuikChange, Stratagene)^15, 47^. The expression and purification of the visual receptors were performed as described previously^15^ with slight modifications. Briefly, plasmids encoding the WT and G90V mutant were expressed in transiently transfected COS-1 cells at 85% confluence by chemical transfection using PEI reagent (100 µL at 1 mg/mL) with 30 µg of plasmid DNA per 145 cm plate. For Q treatment, Q was added to the cell culture medium, from a stock solution in dimethyl sulfoxide (DMSO), to a final concentration of 1 µM Q and 0.25% DMSO. After 48 h, the cells were harvested and the medium was removed. To completely remove the culture medium and Q excess, the cells were washed twice with 15 mL of buffer A. Opsins were subsequently regenerated with 15 µM of 11CR or 9CR in buffer A by overnight incubation at 4°C. Then cells were solubilized, using 1% DM with 100 µM PMSF and protease inhibitors cocktail, by shaking 1 h at 4°C, followed by ultracentrifugation for 30 min at 35000 rpm. WT and G90V mutant were immunopurified from the supernatant using sepharose coupled to rho-1D4 antibody. The resin was washed with buffer B and the bound pigments were subsequently eluted with buffer B containing 100 µM 9-mer peptide. All procedures were performed in the dark or under dim-red light and the samples were always kept on ice.

#### UV-visible spectral characterization

For spectroscopic characterization of Rho samples, a Varian Cary 100 Bio spectrophotometer (Varian, Australia) was used. Temperature was controlled by a peltier accessory equipped with a water-jacketed cuvette holder connected to a circulating water bath. All spectra were recorded in the 250 nm–650 nm range with a bandwidth of 2 nm, a response time of 0.5 s and a scan speed of 400 nm/min.

#### Photobleaching and acidification of purified Rho

Samples were illuminated with a 150-watt Dolan-Jenner MI-150 power source, equipped with an optic fiber guide and using a 495 nm cut-off filter, for 90 s to ensure complete photoconversion to the 380 nm absorbing species. Acidification was carried out, immediately after photobleaching, by the addition of 2N H_2_S0_4_ which yields a pH ~2.0 and the absorption spectrum was subsequently recorded. The reprotonated Schiff base caused by acidification shifts the A_λmax_ to 440 nm.

#### Thermal stability

Thermal stability of Rho was studied by monitoring the decrease of A_max_ of the visible spectral band as a function of time at 48 °C. Spectra were recorded every 5 min and half-life times were determined by fitting the experimental data to single exponential curves.

#### Chemical stability

A solution of 1 M hydroxylamine hydrochloride (adjusted to pH 7) was added to dark-adapted samples in a spectroscopic cuvette (final concentration of 50 mM), and successive spectra were recorded every 5 min to monitor the decrease of A_λmax_ and formation of retinaloxime. The reactions were carried out in the dark at 20 °C. The initial rate was obtained by a linear regression fitting the first data points.

#### Rho regeneration

For the regeneration experiments, 2.5-fold molar excess of 11CR or 9CR (stock solution in ethanol) was added to dark adapted samples in the spectroscopic cuvette and thoroughly mixed. Immediately after, the sample was illuminated with a yellow cut-off filter (>495 nm) to avoid photobleaching of the free retinal, and successive spectra were registered every 10 min in the case of WT and every minute in the case of G90V mutant at 20 °C in the dark until no further increase in A_max_, in the visible region, was observed.

#### Meta II decay

Meta II decay experiments were performed on a QuantaMaster 4 spectrofluorometer (Proton Technology International) equipped with a TLC50 cuvette holder peltier accessory, for temperature control. Initially, the Trp fluorescence of a dark-adapted sample was recorded at 20 °C until a steady base line was obtained. After that, the sample was illuminated for 30 s with a 150-watt Dolan-Jenner MI-150 power source using a cut-off filter (>495 nm) and the fluorescence intensity was monitored until it reached a plateau. All fluorescence spectra were carried out by exciting the samples for 2 s at 295 nm, using a slit bandwidth of 0.5 nm, and blocking the excitation beam for 28 s with a beam shutter to avoid photobleaching of the sample. Trp emission was monitored at 330 nm with a slit bandwidth of 10 nm. The half-life time (t_1/2_) of the fluorescence increase was fit to a single exponential function.

#### Transducin activation assay

Transducin activation was monitored with a radionucleotide filter binding assay. In this assay, GTPγ ^35^S uptake by purified transducin, from bovine retinas, upon binding to activated Rho, was measured. The assay were performed by mixing 10 nM of WT or mutant with 500 nM of transducin in Gt buffer containing 5% of glycerol, 2.5Mm DTT and 5 µM of [S^35^]GTPγS (1250 Ci/mMol)- The reaction was initiated by the addition of the sample in the dark and filtrated after different incubation times (every 4 minutes), either in the dark (0, 4 and 8 min) or after illumination (12, 16, 20, 24 and 28 min) to determine the amount of bound [S^35^]GTPγS.

#### Molecular modeling studies

Docking studies were carried out to identify prospective binding sites of Q on opsin and Rho with 11CR or 9CR bound. For this purpose, the crystallographic structures of opsin (entry 3CAP)^48^, Rho-11CR (entry 1GZM)^49^, Rho-9CR (entry 2PED)^50^, and MetaII (entry 3PXO^51^) were retrieved from the Protein Data Bank. The structures were prepared for docking studies (optimization of hydrogen bonds, protonation states etc.) using the protein preparation wizard tool of the Schrodinger software. Prior to molecular docking studies, all three receptors were examined in order to identify energetically favorable regions for prospective ligand binding. For this purpose, we used the Schrodinger site recognition software SiteMap which identifies binding sites suitable for occupancy by hydrophobic groups or by ligand hydrogen-bond donors, acceptors, or metal-binding functionality from an analysis of size, functionality, and extent of solvent exposure. Subsequently, the structure of Q used in the present study was downloaded from the PubChem website and prepared for docking studies using the LigPrep tool, also from Schrodinger.

#### Data analysis

Results were reported as the mean value ± standard error (SE) obtained in independent purifications (n = 3) and analysed using SigmaPlot 12.5 (Systat Sofware Inc.). Statistical significances were determined by unpaired two-tailed Student’s t-test using. P values < 0.05 were considered to be statistically significant (expressed in the figures with asterisks [*]).

### Data availability

The datasets generated during and/or analysed during the current study are available from the corresponding author on reasonable request.

## Electronic supplementary material

Supplementary information
