# Supplementary material for: Flavonoid allosteric modulation of mutated visual rhodopsin associated with retinitis pigmentosa
Source: Sci Rep. 2017 Sep 11;7:11167. doi: 10.1038/s41598-017-11391-x (PMC5593859; doi:10.1038/s41598-017-11391-x)
Supplement: Supplementary file 1 — Supplementary information [file 41598_2017_11391_MOESM1_ESM.pdf]

Flavonoid allosteric modulation of mutated visual rhodopsin associated with retinitis pigmentosa

María Guadalupe Herrera-Hernández, Eva Ramon, Cecylia S. Lupala, Mercè Tena-Campos, Juan J. Pérez and Pere Garriga.

METHODS

Western blot

For electrophoresis, a Bio-Rad Mini-PROTEAN 2 gel running apparatus was used. A vertical gel was prepared consisting on a separating gel with a degree of crosslinking of 12%, and a stacking gel with low degree of crosslinking (5%). The immunopurified receptors were normalized to equal amounts and mixed with protein loading buffer 4x (Tris 0.0625 M, 2% SDS, 10% glycerol, 0.4M DTT and 0.1% bromophenol blue). The prepared samples and 6 µl of the protein molecular weight marker were loaded into the corresponding wells and the gel was run at 100V for 2 h. The gel was stained overnight using Generon quick Coomassie stain. The stained gels were distained with water until the protein bands could be visualized.

After separating the proteins using SDS-PAGE, the proteins from the gel were transferred onto a nitrocellulose membrane using the *Trans-Blot* SD Semi-Dry Transfer Cell (Bio-Rad). For opsins immunodetection, Rho-1D4 (1:10000 in TBS (8g NaCl, 1.121g Tris, 0.4 ml HCl in 1 L ddH_2O, pH 8.0) buffer) primary antibody and goat anti-mouse secondary IgG antibody (1:5000 in TBS buffer) were used.

Quercetin (Q) identification by HPLC-ESI-MS/MS

For Q extraction, 200 µl of 80% ethanol acidified with 0.1% formic acid was added to the sample. The mixture was vortexed for 1min and then sonicated for 5 min on ice.

After centrifugation at 4000rpm for 20 min at 4°C, the supernatant was collected and evaporated to dryness under a steam of nitrogen gas. The sample was redissolved in 200 µl of 0.1% formic acid. After filtration with 4mm 0.45 µm PTFE syringe filters, 20 µl of the resulting filtrate was injected into the HPLC-MS/MS.

For the HPLC-ESI-MS/MS analysis, an Agilent Technologies 1100 HPLC apparatus equipped with autosampler and column oven (30°C) and coupled to an API 4000 triple-quadrupole mass spectrometer with a TurboIon spray source used in negative mode was used to identify Q. Chromatographic separation was achieved on a Luna C18 (50 x 2.0 mm, 5 µm) from Phenomenex column and a precolumn C18 (4x3 mm i.d.). The mobile phase was water (A) and acetonitrile (B) with 0.1% formic acid in both solvents. An increasing linear gradient (v/v) of B was used as follows: at time =0, 5% of B, from 5% to 18% B in 10 min, from 18% to 100% B in 13 min, 100% B for 1 min and from 100% to 5% of B in 15 min, followed by a 5 min re-equilibration step at a constant flow rate of 0.4 ml min^{-1}. The TurboIon spray source settings were as follows: capillary voltage, -4000V; nebulizer gas (N2), 10 (arbitrary units), curtain gas (N2), 12 (arbitrary units) drying gas (N2) heated to 400°C and introduced at a flow rate of 8000 cm^3 min^{-1}. Full-scan data were acquired by scanning from m/z 100 to 800 in profile mode using a cycle time of 1s.

Molecular modeling studies

Docking studies of Q using Glide (Schrödinger) were carried out on the crystallographic structure of rhodopsin (Rho) bound to 11-cis-retinal and to Rho bound to 9-cis-retinal retrieved from the Protein Data Bank. The structures were prepared for docking studies (optimization of hydrogen bonds, protonation states, etc.) using the protein preparation wizard tool of the Schrodinger software. The structure of Q used in the present study

was downloaded from the PubChem website and prepared for docking studies using the LigPrep tool, also from Schrodinger.

RESULTS

Western blot

The WT (A) and G90V (B) mutant were characterized by Western blot (Fig. S1). A sample of purified Rho from bovine retinas was loaded as a control in addition to the protein ladder molecular marker. Rho shows the two characteristics bands corresponding to the monomer and dimer. A characteristic smear typically observed in COS-1 cells, and usually attributed to heterogeneous glycosylation, was observed in all the samples. No differences were observed in the electrophoretic pattern in WT and G90V mutant as a result of Q treatment. In the case of the G90V mutant, a band below that of the monomer band was noticed. This band may correspond to a truncated protein form which is found around 27 kDa [1–3].

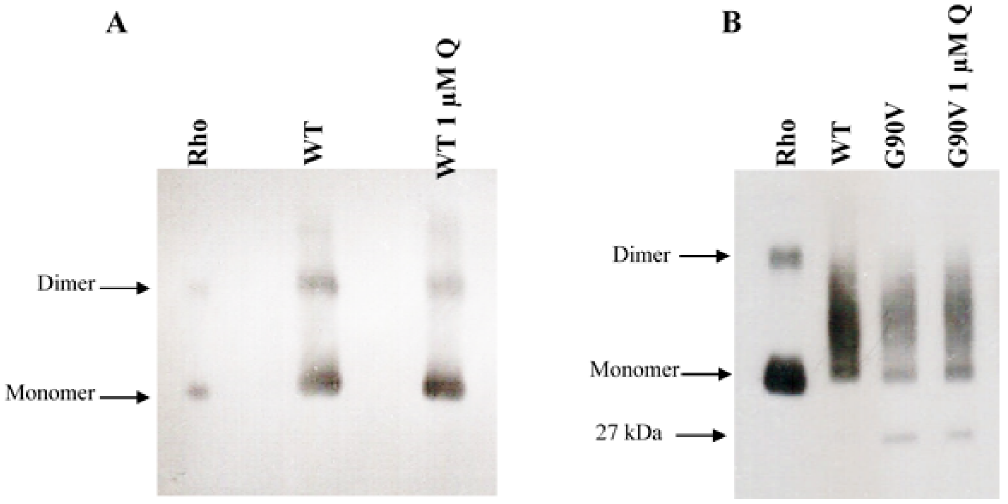

Figure S1. Western blot of WT and G90V mutant with and W/O treatment with 1 μM Q (Q). A, WT with and without Q treatment. **B,** G90V mutant with and without Q treatment.

In the case of the samples obtained from the third elution of G90V 9CR and G90V 9CR-Q at pH 6, they also were analyzed by Western blot (Fig. S2). The electrophoretic pattern of the G90V 9CR-Q mutant showed low intensity in the band corresponding to truncated protein, indicating that Q presence could help in the folding process of this mutant.

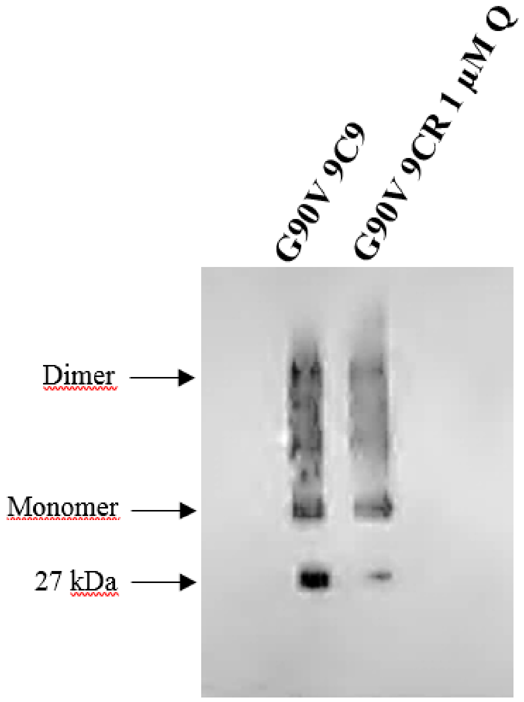

Figure S2. Western blot of immunopurified G90V 9CR and G90V 9CR-Q. The presence of Q decreased the amount corresponding to truncated protein (~27 kDa).

Q identification by HPLC-ESI-MS/MS

The G90V 9CR-Q mutant was analyzed by HPLC-ESI-MS/MS. From the G90V CR-Q mutant sample, Q was isolated as described under *Methods.* For the mass spectrometry study, a Q standard was run at a concentration of 1 ppm. A product ion scan of both the sample and the standard was done (Fig. S3). In this experiment the ions characteristics

of Q could be found in the sample, and the concentration detected was 0.0035 ppm (0.0115 µM).

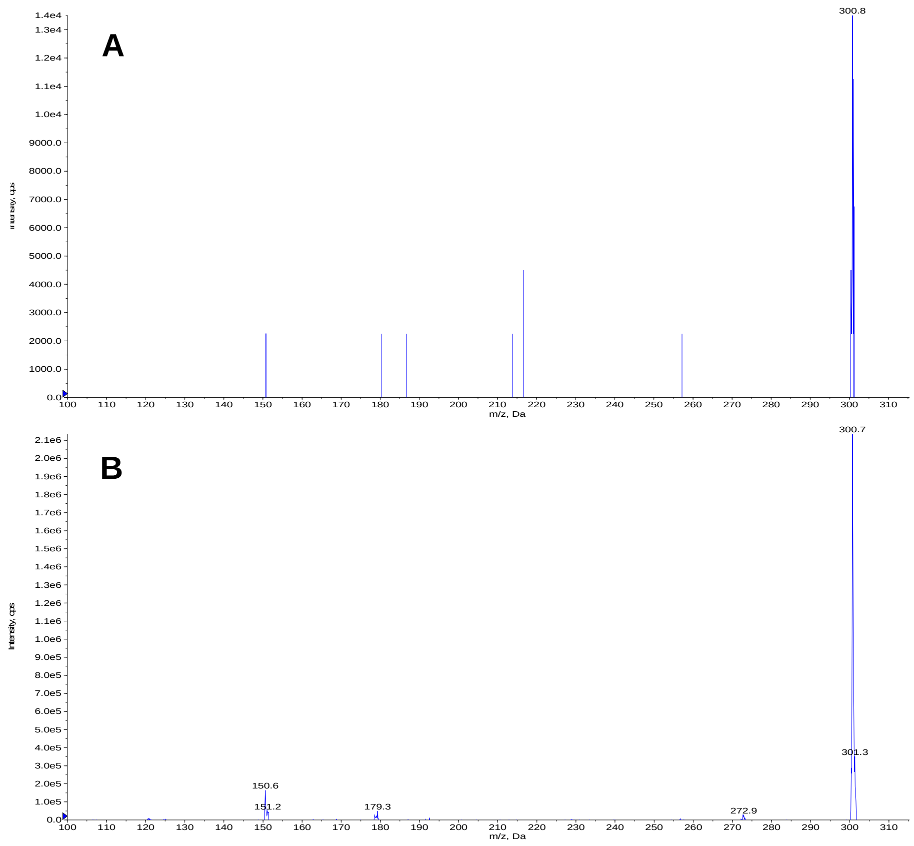

Figure S3. Q identification by HPLC-MS. Mass spectra of Q standard (A) and Q extracted from G90V-9CR-Q (B).

Molecular modeling studies

The superimposition of the structures of Rho bound to 9-*cis*-retinal (Fig. S4 A, in green) and 11-cis-retinal (Fig. S4 A, in blue) show that the ligands do not change significantly the transmembrane region, but the two structures show slight differences in the extracellular loop 2 (ECL2) region (Fig. S4 A). The results of the docking study

revealed that Q binds differentially to both structures. Specifically, it binds to a site involving the ECL2 in isoRho (Fig. S4 B) that is not found on Rho.

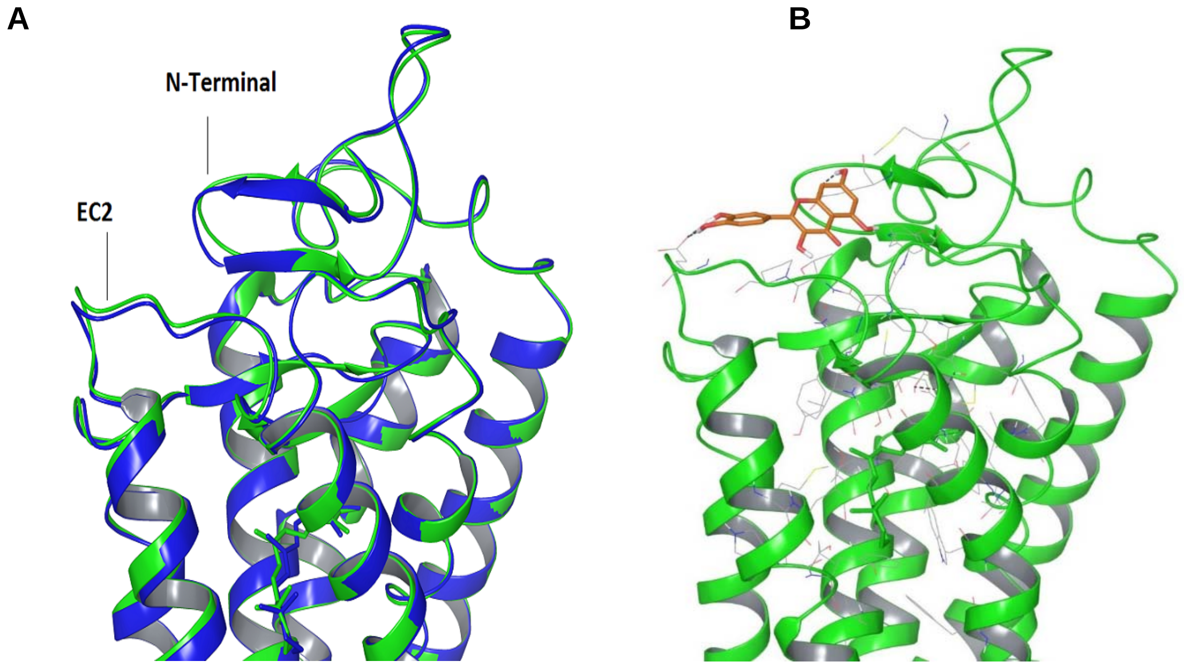

Figure S4. Docking of Q on Rho. A, Superimposition of the structures of Rho bound to 9-*cis*-retinal (in green) and 11-cis-retinal (in blue). **B**, structure of Q (orange) bound to the 9-*cis*-Rho (in green).

References

1. Dong, X., Ramon, E., Herrera-Hernández, M. G. & Garriga, P. Phospholipid Bicelles Improve the Conformational Stability of Rhodopsin Mutants Associated with Retinitis Pigmentosa. *Biochemistry* **54,** 4795–4804 (2015).

2. Fernández-Sampedro, M. A., Invergo, B. M., Ramon, E., Bertranpetit, J. & Garriga, P. Functional role of positively selected amino acid substitutions in mammalian rhodopsin evolution. *Sci. Rep.* **6,** 21570 (2016).

3. Krebs, M. P. *et al.* Molecular mechanisms of rhodopsin retinitis pigmentosa and the efficacy of pharmacological rescue. *J. Mol. Biol.* **395,** 1063–78 (2010).
